# Dental Pulp Stem Cell-Derived Extracellular Vesicles Attenuated Chondrocyte Apoptosis in Early Temporomandibular Joint Osteoarthritis via Regulating Hexokinase 2

**DOI:** 10.3390/biom16040490

**Published:** 2026-03-25

**Authors:** Shengjie Cui, Yu Fu, Xiaotong Yu, Yanning Guo, Jieni Zhang, Xuedong Wang

**Affiliations:** 1 Department of General Dentistry, Peking University School and Hospital of Stomatology & National Center for Stomatology & National Clinical Research Center for Oral Diseases & National Engineering Research Center of Oral Biomaterials and Digital Medical Devices & Beijing Key Laboratory of Digital Stomatology & NHC Key Laboratory of Digital Stomatology & NMPA Key Laboratory for Dental Materials, Beijing 100081, China; 2 Department of Orthodontics, Peking University School and Hospital of Stomatology, Beijing 100081, China; 3 Fourth Clinical Department, Peking University School and Hospital of Stomatology, Beijing 100081, China; 4 Center of Stomatology, Beijing Tsinghua Changgung Hospital, School of Clinical Medicine, Tsinghua Medicine, Tsinghua University, Beijing 102218, China

**Keywords:** temporomandibular osteoarthritis, chondrocyte, extracellular vesicles, apoptosis, glycolysis

## Abstract

Temporomandibular joint osteoarthritis (TMJOA) is a degenerative disease characterized by progressive cartilage destruction, and chondrocyte apoptosis plays a critical role in TMJOA progression. As chondrocytes reside in an avascular microenvironment inside the cartilage matrix, energy production via glycolysis is crucial for their survival. This study investigated the role of the key glycolytic enzyme Hexokinase 2 (HK2) in TMJOA pathogenesis and the therapeutic potential of dental pulp stem cell-derived extracellular vesicles (DPSC-EVs). In a rat experimental TMJOA model induced by monosodium iodoacetate (MIA) intra-articular injection, we observed a significantly decreased expression of HK2 along with cartilage matrix degradation. In the in vitro study, MIA induced chondrocyte apoptosis with caspase-3 activation, accompanied by impaired glycolytic function. Intervention with DPSC-EVs effectively rescued the expression of HK2 within chondrocytes, leading to a notable restoration of cellular glycolysis. Consequently, DPSC-EV treatment markedly attenuated the progression of TMJOA by reducing chondrocyte apoptosis and improved cartilage integrity. Our findings demonstrated that DPSC-EVs represent a promising cell-free therapeutic strategy for TMJOA, exerting their protective effects by targeting HK2, thereby preserving chondrocyte viability and attenuating osteoarthritis development.

## 1. Introduction

Temporomandibular joint osteoarthritis (TMJOA) is a degenerative joint disease characterized by progressive cartilage degradation, subchondral bone remodeling [1,2], and chronic pain, significantly affecting patients’ quality of life [3,4]. The pathogenesis of TMJOA involves a complex interplay between mechanical stress, inflammation, and metabolic dysregulation [5,6,7,8]. Chondrocyte apoptosis is a typical pathological event during cartilage destruction, and our previous study has demonstrated its critical role in promoting subchondral bone resorption by enhancing the chemotaxis of osteoclast precursors [9]. However, it is still challenging to prevent or reverse chondrocyte apoptosis with current clinical therapies.

Chondrocytes, residing in an avascular and hypoxic microenvironment, mainly rely on anaerobic glycolysis for energy production, a process crucial for their survival and function [10]. Hexokinase 2 (HK2) is the rate-limiting enzyme of glycolysis [11], playing a key regulatory role in this metabolic pathway. Recent studies have implicated the dysregulation of glycolysis in the pathogenesis of osteoarthritis [12]. Meanwhile, HK2 could enhance the glycolytic flux in mesenchymal stem cells (MSCs) and orchestrate a crosstalk with immune cells that drives traumatic force-related OA [13]. The current evidence indicates an intricate connection between OA progression and HK2, but the actual role of HK2-driven glycolysis in chondrocytes at the early stage of TMJOA is still unclear.

Current clinical treatment for TMJOA, such as pharmacotherapy and physical therapy, primarily alleviates symptoms but fails to prevent disease progression [14]. Stem cell-based therapies, particularly using mesenchymal stem cells (MSCs) have shown promising effects on cartilage and subchondral bone repair [15,16]. Dental pulp stem cells (DPSCs) and their secretome exert therapeutic effects largely through paracrine signaling [17,18]. The nano-sized extracellular vesicles (EVs) can carry bioactive molecules that modulate immune responses and promote tissue repair [19,20]. Thus, MSC-based therapies provide a new approach to facilitate tissue regeneration for TMJOA.

**Figure 1:**
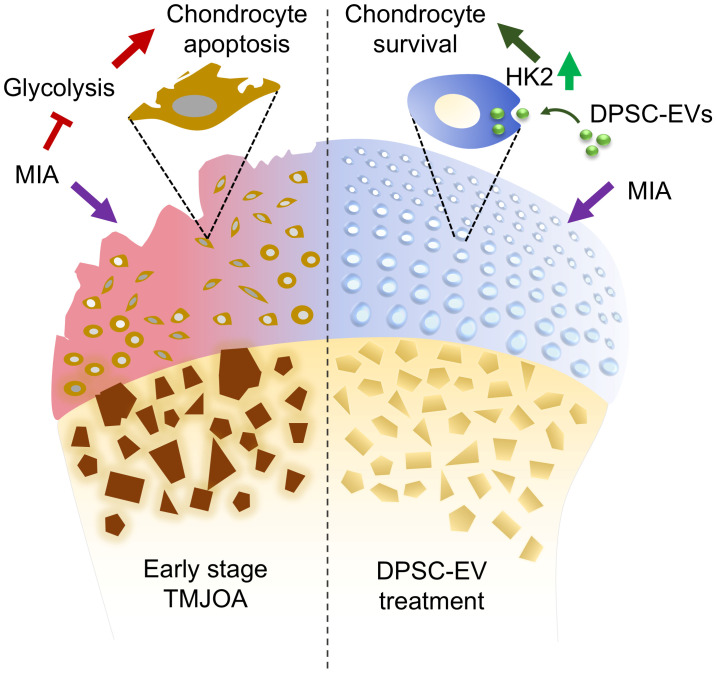
Schematic diagram of the mechanism of DPSC-EV treatment for early stage of experimental TMJOA.

Building upon this foundation, the present study aimed to investigate the role of HK2 dysfunction in TMJOA pathogenesis and the therapeutic potential of DPSC-derived EVs (DPSC-EVs). We confirmed the inhibition of HK2 expression and caspase-3-related cell apoptosis in chondrocytes at the early stage of an experimental TMJOA model, and DPSC-EV treatment significantly rescued this HK2 inhibition, thereby reducing chondrocyte apoptosis and attenuating the progression of TMJOA (Figure 1). The present study systematically evaluated the effects of DPSC-EVs on the HK2-glycolysis-apoptosis axis, offering a metabolic perspective on the mechanism of MSC-based therapy.

## 2. Materials and Methods

### 2.1. Induction of TMJOA

The animal procedures were approved by the Peking University Animal Ethics Committee prior to the initiation of the study (LA2021288). The animal model was established according to our previous studies [21]. Healthy female Sprague-Dawley rats (180–200 g, seven weeks old) were obtained from SiPeiFu Biotechnology Co., Ltd. (Beijing, China). Five rats were housed in one cage. The operators were blinded to the groups.

In the observational experiment, 9 rats were randomly assigned to three groups: the NS group (*n* = 3), MIA 3d group (*n* = 3) and MIA 14d group (*n* = 3). Following intraperitoneal injection of pentobarbital, TMJOA was induced in the experimental groups by the injection of 0.5 mg of monosodium iodoacetate (MIA, Sigma-Aldrich, Saint Louis, MO, USA) dissolved in 50 μL of normal saline (NS) into the upper compartment of the bilateral TMJs with a 27-gauge/0.5-inch needle. The NS group was bilaterally injected with 50 μL of saline only. The MIA 3d group and MIA 14d group were sacrificed 3 days and 2 weeks after MIA injection, respectively. The NS group was sacrificed 2 weeks after the saline injection.

In the therapeutic experiment, 10 rats were randomly assigned to two groups: the MIA + NS group (*n* = 5) and MIA + DPSC-EV group (*n* = 5). The rats received a bilateral intra-articular MIA injection (0.5 mg of MIA dissolved in 50 μL of NS) as described above at the beginning of the experiment. Seven days after MIA injection, 50 μL of saline containing 1 × 10^9^ EV particles was injected into the bilateral TMJs of rats in the MIA + DPSC-EV group, while 50 μL of NS was injected in the MIA + NS group. The rats were euthanized 2 weeks after DPSC-EV/NS injection.

### 2.2. Tissue Harvesting and Histological Staining

The rats were euthanized by CO_2_ asphyxiation at different timepoints after model establishment. For radiological examination, the entire TMJs were carefully removed, fixed in 4% paraformaldehyde for 48 h, and then scanned by micro-computed tomography (CT); for the histological section, the entire TMJs were decalcified with 10% ethylenediaminetetraacetic acid after 48 h fixation, then embedded in paraffin and cut along the sagittal plane to obtain 5 μm thick sections.

For pathological observation, the sections were stained with hematoxylin and eosin (HE) and safranin-O and fast green (SOFG) according to the manufacturer’s instruction (Solarbio, Beijing, China). To evaluate the cartilage destruction during TMJOA, a modified Osteoarthritis Research Society International (OARSI) scoring system of histological staining was chosen, as previously described for TMJOA. Blind scoring was performed by two independent observers, and statistical differences between groups were analyzed [22].

For immunohistochemistry (IHC) staining, the sections were blocked with 5% BSA and incubated with primary antibodies against HK2 (1:100, Abcam, Cambridge, UK) and caspase-3 (1:200, Abcam, Cambridge, UK) at 4 °C overnight. After washing twice with PBS, add HRP-conjugated secondary antibodies for incubation, and then visualize the staining results with diaminobenzidine. In the observational experiment, the sections used for semi-quantitative analysis were obtained from three rats from each group. In the therapeutic experiment, the sections used for semi-quantitative analysis were obtained from five rats from each group. Semi-quantitative counting was performed by researchers blinded to the groups, and data collection and statistical analysis were completed by independent observers.

### 2.3. Micro-CT Scanning

All the samples were scanned with a SkyScan 1174 microcomputed tomography system (micro-CT, 53 kV and 810 μA, Bruker, Billerica, MA, USA). The images were reconstructed and the structural changes in the TMJ of the rats were analyzed using CTan (version 1.16) and CTvox software (version 3.2) (Bruker) according to a previous study [17].

### 2.4. Isolation and Culture of Condylar Chondrocytes

Primary chondrocytes were obtained from the TMJs of three-week-old female rats. The cartilage layer of the condyle was dissected and digested with 0.25% trypsin (Hyclone, South Logan, UT, USA) for 10 min, followed by 0.25% type II collagenase (Sigma-Aldrich, Saint Louis, MO, USA) for 1.5 h. The chondrocytes were then resuspended and cultured in Dulbecco’s modified Eagle’s medium (DMEM, Hyclone, South Logan, UT, USA) containing 10% fetal bovine serum (Hyclone, South Logan, UT, USA) and 100 U/mL of penicillin/streptomycin (Thermo Fisher Scientific, Waltham, MA, USA) under 5% CO_2_ at 37 °C. The chondrocytes at passage 2 were used in the following experiments. To observe the morphology of chondrocytes, observation was performed using an optical microscope. The chondrocytes were fixed with 4% paraformaldehyde for 20 min and stained with Toluidine Blue according to the manufacture’s instruction with the TB kit (Solarbio, Beijing, China).

For apoptosis induction, the condylar chondrocytes were treated with MIA (0, 20, and 100 ng/mL) for 24 h.

### 2.5. Isolation and Culture of DPSCs

The procedures were approved by the Ethics Committee of the Peking University School of Stomatology (PKUSSIRB-202392139). Human DPSCs were obtained from the dental pulp tissue from extracted premolars or wisdom teeth of healthy donors as previously described [17]. After being digested in 3 mg/mL of type I collagenase (Thermo Fisher Scientific, Waltham, MA, USA) and 4 mg/mL of Dispase (Roche, Basel, Switzerland) at 37 °C for 1 h as previously described [23], the cell suspension was cultured with alpha modification of Eagle’s medium (α-MEM, Hyclone, South Logan, UT, USA) containing 15% fetal bovine serum (FBS, Hyclone, South Logan, UT, USA), 2 mM of L-glutamine (Thermo Fisher Scientific, Waltham, MA, USA), and 100 U/mL of penicillin/streptomycin (Thermo Fisher Scientific, Waltham, MA, USA) at 37 °C with 5% CO_2_.

### 2.6. Isolation and Characterization of DPSC-EVs

To acquire DPSC-EVs, DPSCs at passage 4–5 (at 80% confluency) were cultured in a medium containing exosome-depleted FBS for 48 h. The preparation method of exosome-depleted FBS was as follows: FBS was sequentially centrifuged at 2000× *g* for 10 min, 10,000× *g* for 40 min, and 120,000× *g* for 6 h, after which the supernatant was collected [24]. The culture supernatant was collected and centrifuged at 300× *g* for 10 min, 3000× *g* for 10 min, 20,000× *g* for 30 min, and 120,000× *g* for 70 min to collect the extracellular vesicles [25].

**Figure 2:**
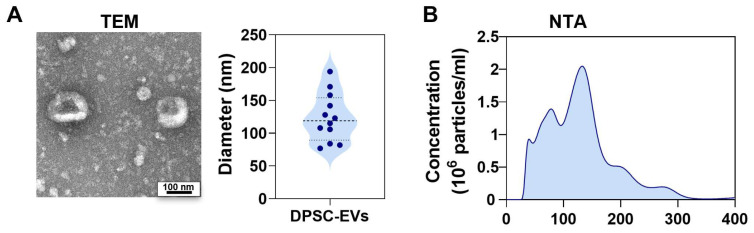
The identification of DPSC-EVs. (**A**) The representative image of DPSC-EVs observed by TEM and semi-quantitative analysis. (**B**) The distribution and counting of DPSC-EVs by nanoSight NTA with 2 peaks of 75 nm and 130 nm.

For the transmission electron microscopy (TEM) observation, freshly isolated EVs derived from 1 × 10^6^ human DPSCs were fixed with an equal volume of 2.5% glutaraldehyde for 20 min at room temperature. Then, 10 µL of the fixed EV suspension was loaded onto formvar–carbon coated copper grids and allowed to adsorb for 20 min at room temperature. After washing with distilled water, the EVs were negatively stained with 2% uranyl acetate for 1 min. Samples were observed using a transmission electron microscope (JEOL, Tokyo, Japan) operating at 100 kV. The TEM results showed that DPSC-EVs exhibited a round shape with an average diameter of 124 ± 36.7 nm (Figure 2A).

For nanoparticle tracking analysis (NTA), the freshly isolated EVs were diluted in PBS to an appropriate concentration range (approximately 10^8^–10^9^ particles/mL) to ensure optimal counting accuracy. The diluted samples were loaded into a 1 mL sterile syringe and injected into the NanoSight system (Malvern Panalytical, Malvern, England), and the mean size (nm) and concentration (particles/mL) of the EVs were calculated. All measurements were performed in triplicate for each sample. The main peaks of particle diameter distribution were located at 75 nm and 130 nm by nanoSight NTA (Figure 2B). Taken together, DPSC-EVs predominantly consist of exosomes [26].

### 2.7. Uptake Assay of DPSC-EVs

DPSC-EVs applied in the present study were freshly isolated without being frozen and thawed. For labeling, PKH-26 (Sigma-Aldrich, Saint Louis, MO, USA) was used for EV labeling according to the instructions, with minor modifications of the manufacture as preciously described [27,28]. Briefly, purified EVs were resuspended in Diluent solution and mixed with PKH-26 dye (4 µM final concentration) for 5 min at room temperature protected from light. The labeling was stopped by adding an equal volume of EV-depleted FBS. Unbound dye was removed by ultracentrifugation at 120,000× *g* for 70 min at 4 °C with sucrose, and the EV pellet was washed once with PBS. The labeled EVs were resuspended in PBS. For co-culture, DPSC-EVs were added into the medium at the final concentration of 2 × 10^8^ particles/mL.

### 2.8. Immunofluorescence Staining

For immunofluorescence staining, cells were incubated with anti-ACAN (1:100; Abcam, Cambridge, UK), anti-COL2 (1:100; Santa Cruz Biotechnology, Paso Robles, CA, USA), anti-F-actin (1:200; Solarbio, Beijing, China), anti-cleaved caspase-3 (1:100; Abcam, Cambridge, UK) and anti-HK2 (1:200; Abcam, Cambridge, UK) primary antibodies overnight at 4 °C. Next, the samples were incubated with secondary antibodies conjugated with FITC or Rhodamine (1:200; Solarbio, Beijing, China) for 1 h at room temperature. After washing with PBS, the samples were mounted with mounting media containing 4′,6-diamidino-2-phenyl-indole (DAPI) for nuclei staining and then observed with a Zeiss LSM 710 laser scanning confocal microscope (Carl Zeiss, Jena, Germany).

### 2.9. Western Blotting Analysis and Quantitative Real-Time Polymerase Chain Reaction

Total proteins of chondrocytes were obtained using a protein extraction kit (RIPA Cocktail; Thermo, Waltham, MA, USA), separated by SDS-PAGE, and transferred onto polyvinylidene difluoride (PVDF) membranes (Millipore, Burlington, MA, USA). The membranes were blocked with 5% non-fat milk in Tris-buffered saline containing 0.1% Tween-20 (TBST) for 1 h, then incubated with anti-HK2 (1:1000; Affinity, Changzhou, China), anti-cleaved caspase-3 (1:1000; Abcam, Cambridge, UK), and anti-β-actin (1:5000, ZSGB-BIO, Beijing, China) primary antibodies. The blots were then incubated with HRP-conjugated secondary antibodies for 1 h at room temperature. Protein bands were visualized using an enhanced chemiluminescence (ECL) detection system. Quantitative analysis was performed with ImageLab software (version 5.0). Original images of all Western blotting are displayed in Figure S1.

Quantitative real-time polymerase chain reaction (qRT-PCR) was applied to examine the expression of *Hk2*, with β-actin serving as the housekeeping gene. Total RNA was extracted by TRIzol reagent (Invitrogen, Waltham, MA, USA). cDNA was synthesized with the SuperScript^®^ III One-Step RT-PCR System with Platinum^®^ Taq High Fidelity (Invitrogen, USA). qPCR was performed on a 7500HT Fast Real-Time PCR machine (Applied Biosystems, Waltham, MA, USA) using SYBR Green (Invitrogen, USA). The primers were designed using Primer Premier 5.0 software and commercially synthesized. The primer sequences were as follows: *Hk2* (forward sequence: 5′-CCAGCAGAACAGCCTAGACC-3′, reverse sequence: 5′-AGATGCCTTGAATCCCTTTG-3′) and *β-actin* (forward sequence: 5′-TGACAGGATGCAGAAGGAGA-3′, reverse sequence: 5′-TAGAGCCACCAATCCACACA-3′).

### 2.10. Annexin-V/PI Assay and Flow Cytometry

Flow cytometry was employed to quantitatively analyze the changes in cell apoptosis via the FITC-Annexin V/PI double-fluorescence labeling method. After collecting chondrocytes, the cells were labeled using the Annexin V-FITC Apoptosis Detection Kit (Solarbio, Beijing, China) in accordance with the manufacturer’s instructions. Subsequently, detection was performed using a flow cytometer (BD Accuri C6 Plus, BD Biosciences, Franklin Lakes, NJ, USA), and the data were analyzed with CFlow Plus software (version 1.0).

### 2.11. Glycolysis Stress Assays

For the glycolysis stress test, extracellular acidification rate (ECAR) measurements were measured using the Seahorse XFe24 Analyzer. Condylar chondrocytes were plated in XF24 cell culture microplates (Agilent, Santa Clara, CA, USA) at a density of 4 × 10^4^ cells/well after being treated with MIA (100 ng/mL) and DPSC-EVs (2 × 10^8^ particles/mL); the cells were then cultured in a CO_2_-free incubator at 37 °C using hippocampal XF assay medium free of glucose prior to analysis. Then, the basal level of ECAR and ECAR sequentially stimulated by glucose (10 mM), oligomycin (1.5 μM), and 2-deoxyglucose (2-DG; 50 mM) were evaluated.

### 2.12. Statistical Analysis

The statistical analysis was conducted with the SPSS 24.0 program. All the data were averaged and expressed as mean ± SD. For Western blotting, glycolysis, qPCR and the semi-quantitative analysis of histological staining and micro-CT, Student’s *t*-test (between two groups) and one-way ANOVA with Tukey’s multiple comparisons test (among three groups) were applied. *p* values of < 0.05 were considered statistically significant.

## 3. Results

### 3.1. Cartilage Matrix Degradation Along with HK2 Inhibition at the Early Stage of TMJOA

**Figure 3:**
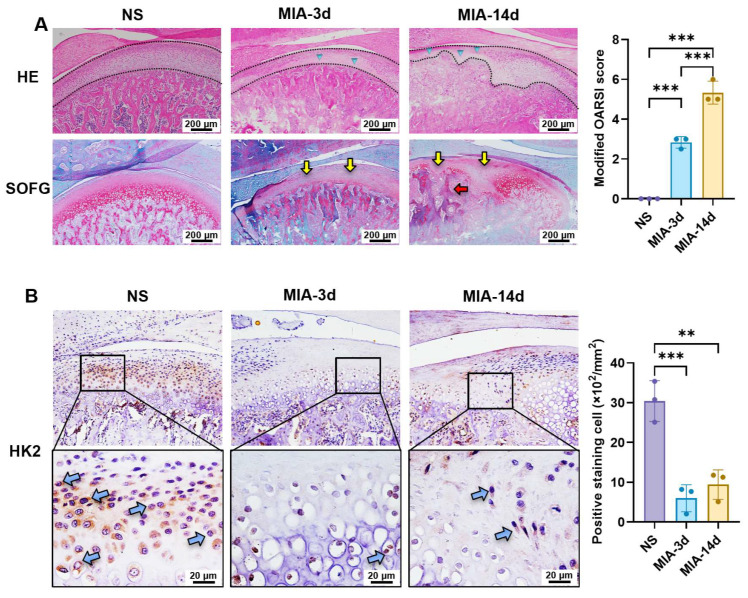
MIA induced cartilage destruction with decreased HK2 expression at the early stage of TMJOA. (**A**) Representative images of histological staining among different groups and semi-quantitative analysis using the modified OARSI score system. Blue triangles: acellular zone; Yellow arrows: loss of matrix staining; Red arrow: thickened trabecula. (**B**) Representative images and semi-quantitative statistics of IHC staining among different groups. Blue arrows: positively stained cells. **: *p* < 0.01, ***: *p* < 0.001.

As demonstrated in our previous studies, MIA induces OA-like pathological changes in the temporomandibular joint [21,29]. In the present study, we established an experimental TMJOA rat model by MIA intra-articular injection. We observed the histological changes in the condyle at different timepoints using HE and SOFG staining. In the NS-injected control group, the chondrocytes were regularly arranged, with distinct cell morphology transitioning from flattened cells in the superficial layer to the mature chondrocytes and further to hypertrophic chondrocytes in the deep zone (Figure 3A). The trabecular bone was evenly distributed and well-formed. SOFG staining exhibited a uniform matrix staining of the cartilage layer. In contrast, the MIA-treated groups manifested typical OA-like cartilage degradation and subchondral bone destruction. At 3 days after MIA injection, the HE staining depicted a significantly reduced thickness of the cartilage layer (black dashed line), along with acellular zones in the cartilage (blue triangles). Meanwhile, SOFG staining showed diminished matrix staining in the middle zone (yellow arrows). With prolonged modeling time, expanded acellular areas, irregular loss of matrix staining and thickening of trabecular bone (red arrow) were observed. The modified OARSI scores were significantly increased, indicating that the physiological contour of the subchondral bone was no longer maintained.

To evaluate whether the expression of HK2 was associated with cartilage destruction, we performed IHC staining. The results showed a large number of positively stained cells (blue arrows) in the middle zone of the cartilage in the NS group (Figure 3B). In contrast, the MIA-3d groups showed a significant reduction in HK2-positive cells, and this trend persisted up to 14 days after MIA injection, confirming the inhibition of HK2 expression in chondrocytes at the early stage of TMJOA.

### 3.2. MIA-Induced Chondrocyte Apoptosis and HK2-Related Glycolysis In Vitro

**Figure 4:**
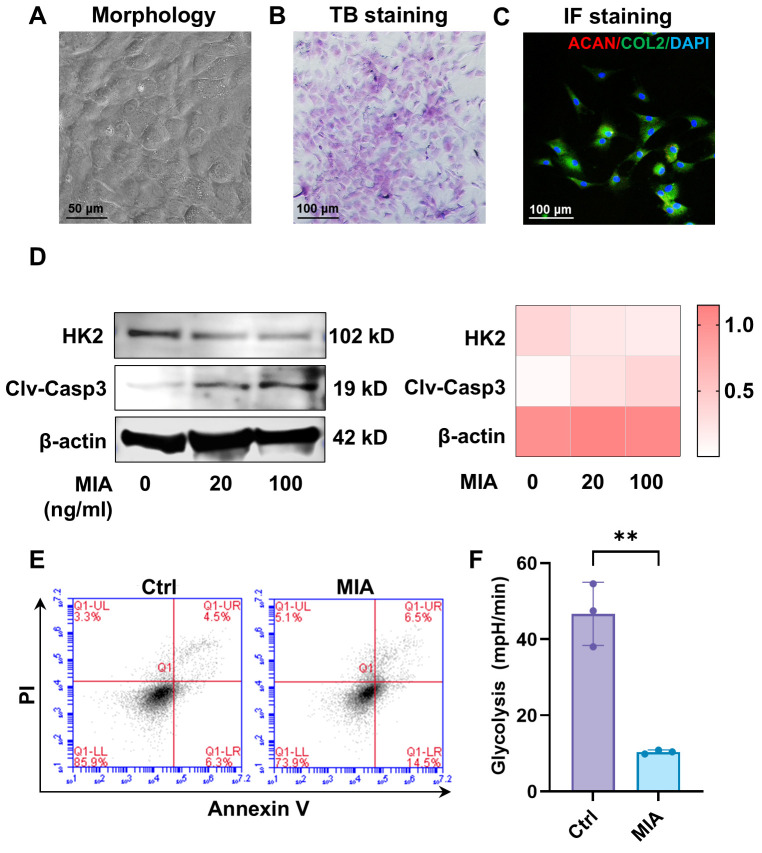
The biological effects of MIA on condylar chondrocytes. (**A**–**C**) The identification of primary condylar chondrocytes by cell morphology (**A**), toluidine blue staining (**B**) and immunofluorescence staining of collagen type II and aggrecan (**C**). (**D**) Western blotting analysis and relative expression statistics of HK2 and cleaved caspase-3 after MIA administration. (**E**) Flow cytometry analysis of chondrocyte apoptosis using an FITC-annexin V/PI kit. (**F**) Glycolytic function of condylar chondrocytes assessed by glycolytic stress test measuring ECAR. TB: toluidine blue; IF: immunofluorescence; Clv-Casp3: cleaved caspase-3. **: *p* < 0.01.

To evaluate the biological effects of MIA on the chondrocytes, we dissected the condylar cartilage and isolated primary chondrocytes by sequential digestion with trypsin and collagenase type II. The cultured chondrocytes exhibited a polygonal shape (Figure 4A) and positive staining with toluidine blue (TB) (Figure 4B). Meanwhile, we observed double positive staining of collagen type II (COL2) and aggrecan (ACAN) in passage 2 chondrocytes (Figure 4C), confirming that the cultured primary cells maintained chondrocyte characteristics.

To testify the influence of MIA on glycolysis and apoptosis, we first evaluated the expression of HK2, the rate-limiting enzyme of glycolysis, and the activation of caspase-3, the main executioner of caspase-dependent classical apoptosis. The results of Western blotting showed that with increasing concentrations of MIA, the expression of HK2 in condylar chondrocytes significantly decreased (Figure 4D), and the expression of cleaved caspase-3, the active form of caspase-3, was increased. These data indicate a possible relationship between glycolysis and chondrocyte apoptosis.

Annexin V/PI apoptosis assay (Figure 4E) exhibited a significantly increased ratio of Annexin V-positive cells after MIA treatment, demonstrating the pro-apoptotic effects of MIA. Meanwhile, the ECAR from glycolytic stress tests showed significantly decreased glycolysis in chondrocytes after MIA treatment (Figure 4F). Taken together, MIA-induced glycolytic inhibition promoted chondrocyte apoptosis, suggesting that targeting HK2, the first rate-limiting enzyme of glycolysis, may provide a new approach to prevent chondrocyte apoptosis.

### 3.3. DPSC-EVs Rescued MIA-Induced Chondrocyte Apoptosis In Vitro

**Figure 5:**
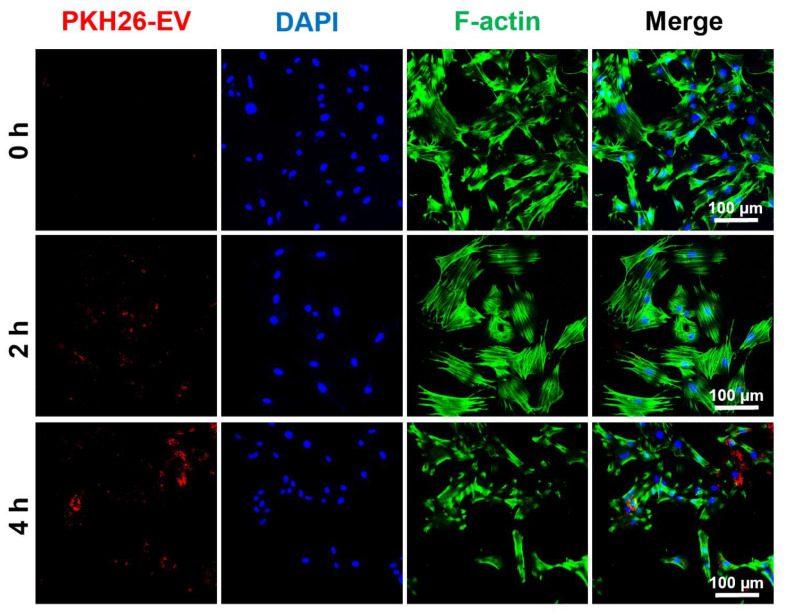
The cellular uptake of DPSC-EVs. Representative immunofluorescence images of the cell uptake of PKH26-labeled DPSC-EVs (red) after incubating with condylar chondrocytes for 2 h and 4 h.

We previously reported the therapeutic effects of DPSCs and their EVs on TMJ arthritis and knee OA, respectively [17,30]. To explore the therapeutic potential of DPSC-EVs on chondrocyte apoptosis, we first evaluate the cell uptake of DPSC-EVs (Figure 5). The results showed that after 2 h of co-culture with chondrocytes, DPSC-EVs began to be internalized into the cells, and after 4 h of incubation, a large number of DPSC-EVs were detected within the condylar chondrocytes. Therefore, we added DPSC-EVs 4 h prior to MIA treatment in subsequent experiments.

**Figure 6:**
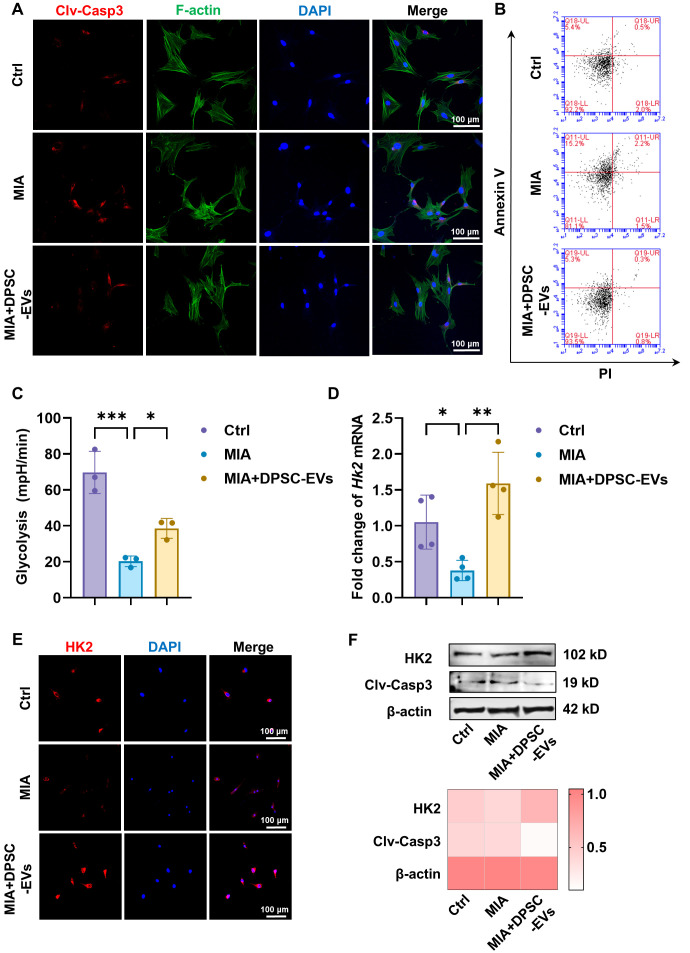
DPSC-EVs rescued MIA-induced chondrocyte apoptosis and HK2-related glycolysis in vitro. (**A**) Representative images of the immunofluorescence staining of cleaved caspase-3. (**B**) Flow cytometry results of FITC-annexin V/PI analysis to evaluate cell apoptosis of condylar chondrocytes. (**C**) Glycolytic function of condylar chondrocytes evaluated by ECAR among three groups. (**D**,**E**) Restored HK2 expression of the MIA + DPSC-EVs group demonstrated by qRT-PCR (*n* = 4) (**D**) and immunofluorescence staining (**E**). (**F**) Western blotting analysis and relative expression statistics of HK2 and cleaved caspase-3 after MIA + DPSC-EVs treatment. Clv-Casp3: cleaved caspase-3. *: *p* < 0.05, **: *p* < 0.01, ***: *p* < 0.001.

We then investigated the effects of DPSC-EVs on chondrocyte apoptosis. The immunofluorescence staining showed significant upregulation of cleaved caspase-3 after MIA treatment, and DPSC-EVs effectively reversed this increase (Figure 6A). The flow cytometry results demonstrated the cell survival of the MIA + DPSC-EVs group with a reduced ratio of annexin V-positive cells compared with the MIA group (Figure 6B). Thus, the administration of DPSC-EVs could effectively reversed MIA-induced chondrocytes apoptosis.

To further testify the role of HK2-related glycolysis of the protective effects of DPSC-EVs, we evaluated the glycolytic activity among different groups, and the results of the MIA + DPSC-EVs group exhibited a significant recovery of glycolytic ECAR compared with the MIA group (Figure 6C). Consistently, both mRNA and protein expressions of HK2 were evaluated by qRT-PCR (Figure 6D) and immunofluorescence staining (Figure 6E), and DPSC-EVs significantly restored the expression HK2. The results of Western blotting (Figure 6F) confirmed the enhanced expression of HK2 accompanied with the inhibition of cleaved caspase-3 after DPSC-EV administration. These molecular biology assays demonstrated that DPSC-EVs effectively ameliorated the MIA-induced HK2 inhibition, thereby restoring intracellular glycolytic function.

### 3.4. DPSC-EVs Attenuated the Progression of TMJOA In Vivo

**Figure 7:**
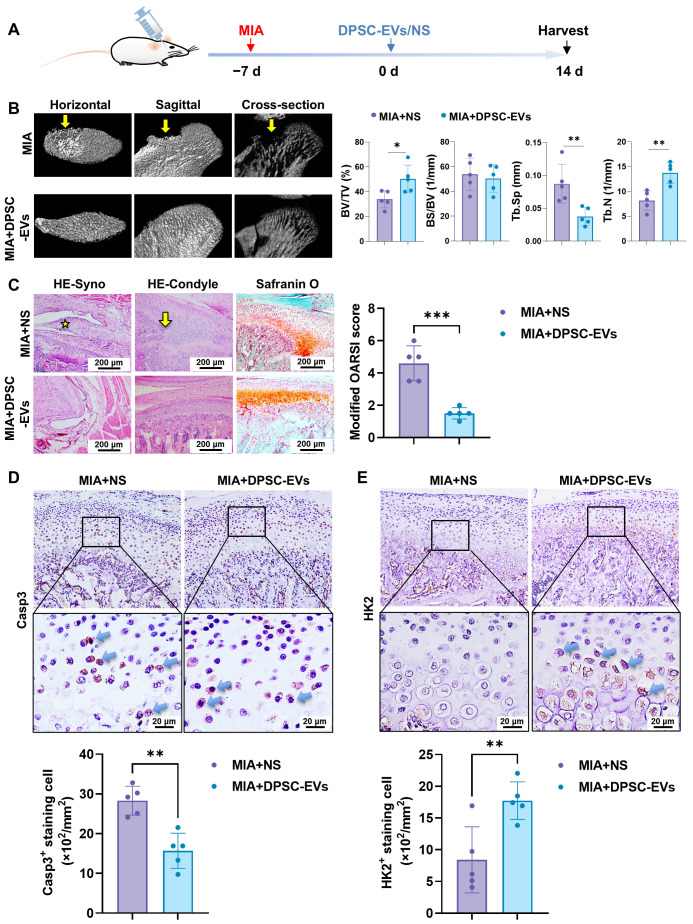
DPSC-EVs improved the radiological and histological changes in TMJOA in vivo. (**A**) Schematic timeline of the TMJOA modeling and treatment procedure. (**B**) Representative images of micro-CT and quantitative statistics. (**C**) Representative images of histological staining from different groups and quantification using the modified OARSI scoring system. Yellow star: multiple layers of synovial lining cells; yellow arrows: abnormal morphology of subchondral bone. (**D**,**E**) Representative images and semi-quantitative statistics of the IHC staining of caspase-3 (**D**) and HK2 (**E**). Casp3: caspase-3. Blue arrows: positively stained cells. *: *p* < 0.05, **: *p* < 0.01, ***: *p* < 0.001.

To further demonstrate the therapeutic potential and the biological mechanism of DPSC-EVs, we next testify the radiological and histological changes in the TMJs after DPSC-EV intra-articular injection in a rat TMJOA model (Figure 7A). Micro-CT scanning showed a substantial defect of the condylar bone on the anterior slope of the condyle as the yellow arrows showed (Figure 7B), while the MIA + DPSC-EVs significantly attenuated the bone destruction of the condyle with increased bone volume/tissue volume (BV/TV) and trabecular bone number (Tb.N) and decreased trabecular separation (Tb.Sp). Hence, the local injection of DPSC-EVs effectively ameliorated the subchondral bone damage and attenuated the progression of TMJOA.

The histological staining further validated the therapeutic effects of DPSC-EVs (Figure 7C). The results of HE staining showed multiple layers of synovial lining cells (yellow star), indicating synovial inflammation in the MIA + NS group, while the MIA + DPSC-EVs group exhibited thinner layers of the synovial tissue. Meanwhile, the abnormal morphology of subchondral bone (yellow arrow) was observed with uneven and discontinuous matrix staining on SOFG staining in the MIA + NS group. In contrast, the physiological morphology of cartilage and subchondral bone was well-maintained with continuous matrix staining and significantly lower modified OARSI scores in the MIA + DPSC-EVs group.

We then testified the expression of caspase-3 and HK2 of the chondrocytes (Figure 7D,E). The results of IHC staining exhibited caspase-3 positive chondrocytes in the middle zone in MIA groups, and the DPSC-EVs significantly reduced the quantities of positively stained cells (Figure 7D). Meanwhile, the expression of HK2 in the MIA + DPSC-EVs group was significantly upregulated compared with the MIA group (Figure 7E), confirming the critical role of HK2 in DPSC-EV therapy.

## 4. Discussion

In the present study, we elucidated a novel mechanism by which DPSC-derived extracellular vesicles mitigated temporomandibular joint osteoarthritis, primarily through rescuing HK2-mediated glycolysis and the subsequent inhibition of chondrocyte apoptosis. The in vitro experiments demonstrated that MIA administration suppressed HK2 expression and glycolytic flux in condylar chondrocytes, thereby activating caspase-3-dependent apoptosis. Crucially, intervention with DPSC-EVs effectively reversed these pathological changes, rescuing HK2 expression, normalizing glycolysis, and reducing chondrocyte apoptosis. These protective effects were further confirmed in a rat TMJOA model, where early DPSC-EV administration significantly attenuated cartilage degradation and subchondral bone destruction, downregulated caspase-3 expression, and upregulated HK2 expression in chondrocytes. Collectively, our data clarify the impairment of HK2-driven glycolysis as a pivotal metabolic deficiency in the early stage of TMJOA and identify DPSC-EVs as a potent therapeutic agent capable of chondrocyte viability protection and joint integrity.

As the development of OA related to multiple complex factors [31], chondrocyte apoptosis plays a central role in driving cartilage degradation and subchondral bone abnormal remodeling during TMJOA pathogenesis [9,32]. Residing in a relatively avascular and hypoxic niche, chondrocytes are fundamentally dependent on anaerobic glycolysis for energy production. Our data suggest that the initial suppression of HK2 and the consequent glycolytic impairment were closely related to chondrocytes apoptosis, thereby initiating the cascade of cartilage and subchondral bone destruction. This finding aligns with the concept that energy failure is a potent trigger for programmed cell death [33]. However, in progressive OA, a metabolic shift characterized by upregulation of glycolytic enzymes has been observed in multiple cell types, including immune cells and synoviocytes [13,34,35]. This apparent paradox reflects the dynamic nature of the metabolic microenvironment at different stages of OA progression.

The use of human MSCs and MSC-derived EVs in rodent xenogeneic models is supported by the low immunogenicity of MSCs, which enables immune evasion and allows for preclinical evaluation of MSC-based therapies [36,37]. While our study demonstrates that DPSC-EVs attenuate chondrocyte apoptosis and restore HK2-mediated glycolysis, we acknowledge that the detailed molecular profiling of these EVs remains a limitation. Although we characterized EVs by TEM and NTA, a comprehensive analysis of their cargo (such as miRNA sequencing or proteomic profiling) was not performed. This limits our ability to identify the key bioactive molecules responsible for chondrocyte survival. Emerging evidence highlights the heterogeneity of MSC-derived EVs and the critical role of their cargo diversity in determining therapeutic outcomes [38]. For instance, DPSC-EVs have been shown to carry specific microRNAs (e.g., miR-140-5p) that regulate cell homing and differentiation [39]. Future studies employing multi-omics approaches will be essential to identify the specific EV components, including miRNAs, proteins, or lipids, that directly modulate the HK2-glycolysis axis and to validate their functional roles. Such investigations would further strengthen the understanding of the mechanisms by which DPSC-EVs exert therapeutic effects in early TMJOA.

We previously reported the direct influence of DPSC-EVs on osteoclast precursors [30], and the present findings demonstrate that DPSC-EVs could exert their therapeutic effects by effectively suppressing the critical apoptotic cascade of chondrocytes. Our results add to current biological mechanisms of MSC-based therapies, providing a comprehensive understanding of DPSC-EV-promoted glycolytic recovery in OA chondrocytes. Future investigations are warranted to clarify the temporal metabolic profile, examining how glycolytic flux and HK2 expression influence energy metabolism, which would offer a more comprehensive therapeutic strategy targeting metabolism. However, the rapid clearance of freely injected EVs may limit the application for translational medicine [40]. Biomaterial-based strategies, such as hyaluronic acid (HA) or collagenous hydrogels are needed to enable the sustained release of therapeutic EVs [41].

Despite the compelling evidence, this study has several limitations. Firstly, we chose to use EV-depleted FBS in the culture medium to ensure good cell viability. However, although we observed an increase in EV-associated markers in the conditioned medium after DPSC culture, this approach does not completely eliminate the confounding effects of residual FBS-derived EVs. Studies have shown that even prolonged ultracentrifugation (18 h) achieves only partial EV depletion (approximately 82%) from FBS [42]. To better elucidate the biological effects of DPSC-EVs, alternative strategies such as ultrafiltration or serum-free culture systems offer preferable options, as they minimize exogenous EV contamination while better preserving MSC functionality [43,44]. Secondly, although we documented a clear recovery of ECAR, the assessment of the overall cellular energy status remains incomplete. In order to clarify the balance between glycolytic and oxidative metabolism, further studies are needed to evaluate mitochondrial oxidative phosphorylation (OXPHOS) and to obtain quantitative measurements of intracellular ATP levels. In addition, the specific causal relationship between HK2 recovery and apoptosis suppression, while strongly correlated, would be more robustly validated using HK2-specific genetic models. Addressing these limitations will significantly deepen our understanding of the metabolic therapy of TMJOA.

## 5. Conclusions

In summary, this study establishes that restoring HK2-mediated glycolysis via DPSC-EVs effectively protects chondrocytes from apoptosis and attenuates early TMJOA progression in the MIA-induced rat model. However, we acknowledge that the MIA model induces OA-like pathology through direct glycolytic inhibition, whereas human OA chondrocytes exhibit a more complex metabolic landscape, often characterized by enhanced glycolysis at the late-stage of OA. Therefore, while our findings provide a new insight that targeting the HK2-glycolysis-apoptosis axis could be a relevant therapeutic strategy, they cannot be directly extrapolated to human TMJOA treatment. These findings demonstrate that maintaining metabolic balance is essential for chondrocyte survival, and provide a basis for designing EV-based therapies that adapt to the different metabolic stages of OA.

## Data Availability

Data is contained within the article and Supplementary Materials.

## Supplementary Materials

The following supporting information can be downloaded at: https://www.mdpi.com/article/10.3390/biom16040490/s1, Figure S1: The original images of all Western blotting images.

## Abbreviations

The following abbreviations are used in this manuscript:

TMJOA	Temporomandibular joint osteoarthritis
HK2	Hexokinase 2
Casp3	Cysteinyl aspartate specific proteinase 3
DPSCs	Dental pulp stem cells
EVs	Extracellular vesicles
MIA	Monosodium iodoacetate
NS	Normal saline
COL2	Collagen type II
ACAN	aggrecan
OARSI	Osteoarthritis Research Society International
ECAR	Extracellular acidification rate
