# Supplementary material for: Dental Pulp Stem Cell-Derived Extracellular Vesicles Attenuated Chondrocyte Apoptosis in Early Temporomandibular Joint Osteoarthritis via Regulating Hexokinase 2
Source: Biomolecules. 2026 Mar 25;16(4):490. doi: 10.3390/biom16040490 (PMC13113402; doi:10.3390/biom16040490)
Supplement: Supplementary file 1 [file biomolecules-16-00490-s001.zip › biomolecules-4133000-supplementary.pdf]

Dental pulp stem cell-derived extracellular vesicles attenuated chondrocyte apoptosis in early temporomandibular joint osteoarthritis via regulating Hexokinase 2

Supplementary Material

Fig 4D

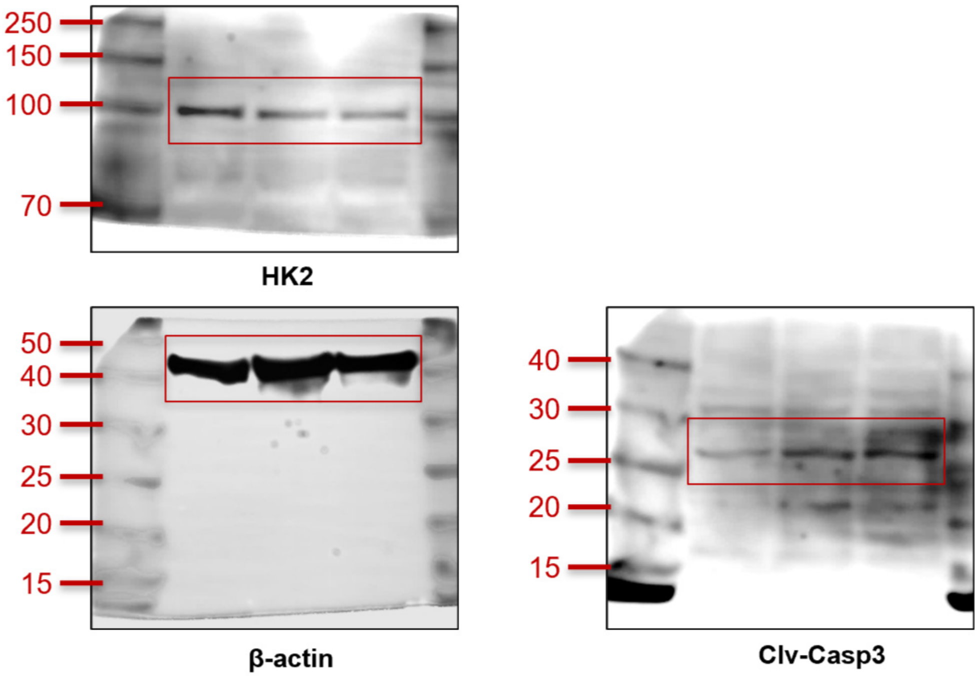

Fig 6F

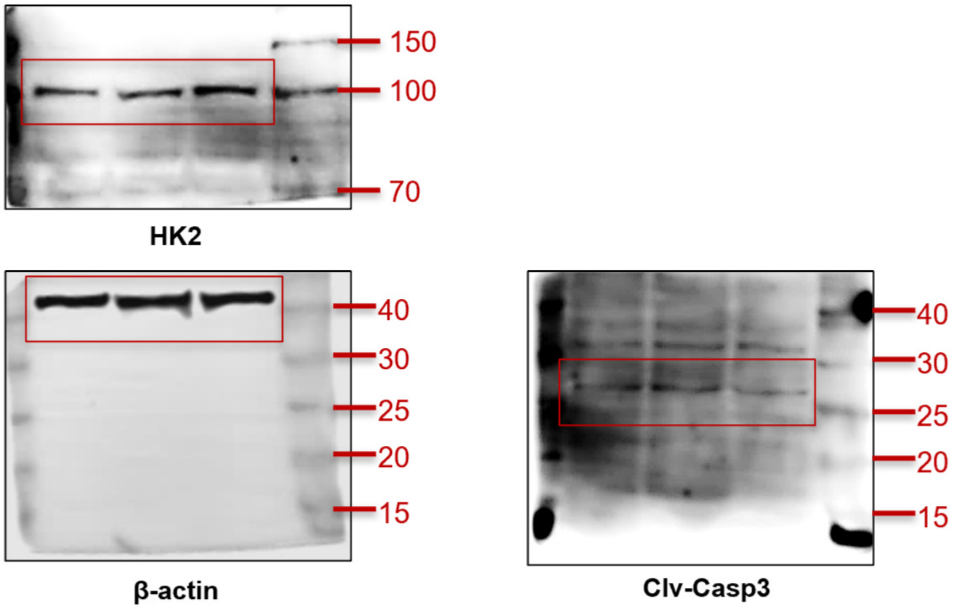

Figure S1. The original images of all western blotting images.
